# A comparison between a new automatic system and Doppler method for obtaining ankle brachial pressures

**DOI:** 10.1186/1757-1146-3-S1-O15

**Published:** 2010-12-20

**Authors:** Jane Lewis, Melinda Hawkins, Phillip Barree, Scott Cawley, Susan Dayananda

**Affiliations:** 1 Cardiff and Vale University Health Board, Cardiff, Wales, UK

## Objectives and relationship to conference themes

The Objective was to develop and validate a quicker and more reliable method of assessing for peripheral arterial disease within the primary care setting.

## Introduction

The prevalence of peripheral arterial disease (PAD) increases with age, affecting 3% under 60 years, rising to >20% over 75 years. Whilst 40% of PAD patients are symptomatic, around 60% are asymptomatic.

PAD is a reliable marker of future vascular disease such as Congestive Heart Disease (CHD) and stroke, and a substantial public health issue. In the UK around 100,000 people are diagnosed every year resulting in 60% of PAD patients dying from MI and 12% from stroke. PAD is present in ~30% of the diabetic population and considered to be a greater risk-factor than neuropathy for foot ulceration.

The purpose of this study is to test the hypotheses that using the new device (Dopplex Ability),

A) the ABPI test can be performed quicker than Doppler,

B) obviates the need to rest the patient and

C) provides results in good agreement with Doppler.

The analysis methods used were Bland Altman agreement plots, equality plots and Pearson correlation.

## Method

A randomized cross-over study design (N=200 subjects, 400 limbs) was chosen so that unbiased comparisons of Dopplex Ability unrested and Doppler, and Dopplex Ability rested and Doppler could be made. The time taken for each test was noted.

## Results

The results show good agreement between unrested Dopplex Ability and Doppler (r=0.894, p<0.05) and rested Dopplex Ability and Doppler (r=0.907, p<0.05). 95% limits of agreement were +0.22 with a bias of -0.062 for unrested Dopplex Ability and Doppler and +0.20 with a bias of -0.044 for rested Dopplex Ability and Doppler. The mean time taken to perform the tests was 7.1 minutes for Dopplex Ability, due to the simultaneous cuff inflation, and 16.5 minutes +15-20mins resting time for Doppler.

## Conclusion

The results show that there is good agreement between the Dopplex Ability and the hand held Doppler. The Ability measurement takes significantly less time than Doppler and obviates the need for a rested patient improving the whole patient experience. The Ability has the potential for PAD screening in the primary care environment with minimal training required due to its ease of use.

